# Inhaled Nitric Oxide at Birth Reduces Pulmonary Vascular Resistance and Improves Oxygenation in Preterm Lambs

**DOI:** 10.3390/children8050378

**Published:** 2021-05-11

**Authors:** Satyan Lakshminrusimha, Sylvia F. Gugino, Krishnamurthy Sekar, Stephen Wedgwood, Carmon Koenigsknecht, Jayasree Nair, Bobby Mathew

**Affiliations:** 1 Departments of Pediatrics, University of California at Davis, UC Davis Children’s Hospital, 2516 Stockton Blvd, Sacramento, CA 95817, USA; swedgwood@ucdavis.edu; 2 Department of Pediatrics, State University of New York at Buffalo, Buffalo, NY 14222, USA; sfgugino@buffalo.edu (S.F.G.); carmonko@buffalo.edu (C.K.); jnair@upa.chob.edu (J.N.); bmathew@upa.chob.edu (B.M.); 3 Physiology and Biophysics, State University of New York at Buffalo, Buffalo, NY 14222, USA; 4 Department of Pediatrics, University of Oklahoma, Oklahoma City, OK 73013, USA; Krishnamurthy-Sekar@ouhsc.edu

**Keywords:** inhaled nitric oxide, resuscitation, prematurity, persistent pulmonary hypertension of newborn, pulmonary vascular resistance, hypoxic pulmonary vasoconstriction

## Abstract

Resuscitation with 21% O_2_ may not achieve target oxygenation in preterm infants and in neonates with persistent pulmonary hypertension of the newborn (PPHN). Inhaled nitric oxide (iNO) at birth can reduce pulmonary vascular resistance (PVR) and improve PaO_2_. We studied the effect of iNO on oxygenation and changes in PVR in preterm lambs with and without PPHN during resuscitation and stabilization at birth. Preterm lambs with and without PPHN (induced by antenatal ductal ligation) were delivered at 134 d gestation (term is 147–150 d). Lambs without PPHN were ventilated with 21% O_2_, titrated O_2_ to maintain target oxygenation or 21% O_2_ + iNO (20 ppm) at birth for 30 min. Preterm lambs with PPHN were ventilated with 50% O_2_, titrated O_2_ or 50% O_2_ + iNO. Resuscitation with 21% O_2_ in preterm lambs and 50%O_2_ in PPHN lambs did not achieve target oxygenation. Inhaled NO significantly decreased PVR in all lambs and increased PaO_2_ in preterm lambs ventilated with 21% O_2_ similar to that achieved by titrated O_2_ (41 ± 9% at 30 min). Inhaled NO increased PaO_2_ to 45 ± 13, 45 ± 20 and 76 ± 11 mmHg with 50% O_2_, titrated O_2_ up to 100% and 50% O_2_ + iNO, respectively, in PPHN lambs. We concluded that iNO at birth reduces PVR and FiO_2_ required to achieve target PaO_2_.

## 1. Introduction

During fetal life, pulmonary vascular resistance (PVR) is high and PaO_2_ levels are low compared to the postnatal period [1]. Oxygen is a potent and specific pulmonary vasodilator and plays an important role in decreasing PVR at birth [1]. After birth, PVR gradually decreases and oxygenation slowly improves over the first minutes of life. Current neonatal resuscitation guidelines recommend the use of 21% oxygen in the delivery room resuscitation of term infants [2]. However, controversy exists as to the optimal resuscitation gas in preterm infants [3]. Recent studies suggest that extremely preterm infants who were first resuscitated with 21% oxygen and titrated up to achieve target SpO_2_ had higher mortality from respiratory failure compared to infants whose resuscitation was initiated with 100% oxygen and titrated down [4]. However, high initial inspired oxygen concentration (>65%) is not recommended during resuscitation of preterm infants due to the risk of oxidative stress [5,6]. Promoting pulmonary vasodilation without excessive supplemental oxygen can potentially facilitate the establishment of gas exchange in the lung without exposing the infant to oxygen toxicity.

**Figure 1:**
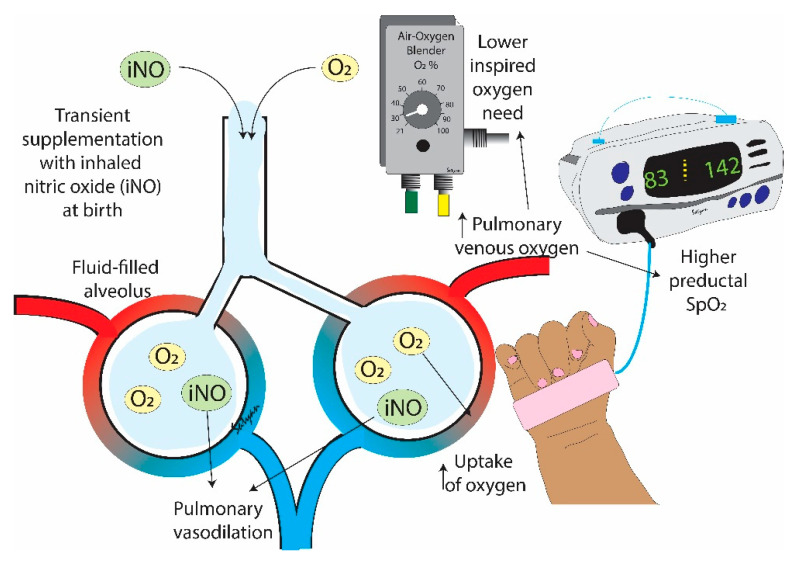
Hypothesis. Newly born, preterm infants have immature lungs filled with liquid. Administration of low concentrations of inspired oxygen alone may not be adequate to achieve target oxygen saturation (SpO_2_). Transient supplementation with inhaled nitric oxide (iNO) during delivery room resuscitation and stabilization can promote pulmonary vasodilation and enhance gas exchange leading to a lower need for inspired oxygen and higher preductal SpO_2_. Copyright Satyan Lakshminrusimha.

Persistent pulmonary hypertension of the newborn (PPHN) [7] is a disorder characterized by elevated pulmonary vascular resistance (PVR), extra-pulmonary right-to-left shunting and hypoxemia. Inhaled nitric oxide (iNO) is a selective pulmonary vasodilator approved by the Food and Drug Administration in term infants with PPHN and acts by increasing cGMP in pulmonary arterial smooth muscle cells (PASMC). We hypothesized that ventilation with iNO at birth would reduce PVR and increase arterial partial pressure of oxygen both in preterm newborn lambs and in PPHN lambs, similar to that achieved by high FiO_2_ (Figure 1). Our overall aim was to evaluate if iNO supplementation resulted in a reduced need for inspired oxygen as being secondary to a decrease in PVR in animal models with and without PPHN.

## 2. Materials and Methods

This study was approved by the University at Buffalo Institutional Animal Care and Use Committee. Time-dated pregnant ewes were procured from New Pasteur farms, Attica, NY. Lambs were delivered by caesarean section at 134 d gestation (term gestation in lambs is 147–150 days). We used the ovine in utero ductal ligation model to induce PPHN. Time-dated pregnant ewes were anesthetized as previously described [8] and fetal ductus arteriosus was ligated at 128 d gestational age [9]. The fetus was then placed back in the uterus for 8 days. On the day of delivery, preterm lambs (with and without PPHN) were partially exteriorized by cesarean section and catheters were placed in the jugular vein and carotid artery to obtain PaO_2_ measurements at different levels of oxygen exposure with and without iNO. The dose of iNO was 20 ppm. We conducted the study in 2 phases:

I. Oxygenation studies: Preterm lambs with and without PPHN were intubated at birth and randomized to be ventilated with 21, 50 or 100% oxygen with or without iNO for 30 min. Preductal arterial gases were drawn every 5 min and recorded.
II. Pulmonary hemodynamic studies: In a subsequent set of experiments, we studied the effect of iNO on oxygenation and pulmonary vascular resistance. These lambs were exteriorized as described previously [8,9]. In addition to the placement of right carotid and jugular lines, we performed a thoracotomy and placed pulmonary arterial and left atrial catheters to measure pressures and a pulmonary arterial flow probe to measure blood flow. The flow probe was placed around the left pulmonary artery in lambs without PPHN to avoid the influence of blood flow through the patent ductus arteriosus (PDA). In lambs with PPHN, the flow probe was placed around the main pulmonary artery as the ductus arteriosus was ligated in utero to induce PPHN.
  a. Based on results from phase I oxygenation studies, preterm lambs without PPHN were exposed to 21% oxygen, titrated oxygen to maintain PaO_2_ between 45 and 80 mmHg and titrated oxygen with iNO at 20 ppm.
  b. In lambs with PPHN, 21% oxygen was avoided as PaO_2_ levels were low with this FiO_2_ in phase I oxygenation studies. PPHN lambs were exposed to 50% oxygen, titrated oxygen to maintain PaO_2_ between 45 and 80 mmHg and titrated oxygen with iNO at 20 ppm for 30 min.
  c. Lambs were effectively anesthetized during the period of instrumentation through the isoflurane inhalant administered to the ewe. The lambs were then delivered and ventilated at the following initial settings: 30 cm H_2_O peak inspiratory pressure, 5 cm H_2_O positive end expiratory pressure, and 40 respirations per minute. Sedation was maintained by administration of an initial propofol bolus (2 mg/kg) followed by a constant rate infusion given to effect. Additional doses of fentanyl at 1–5 mcg/kg were administered as needed for signs of discomfort. Maintenance IV fluid with dextrose and electrolytes was also provided. Arterial blood pressure, heart rate, and pulse oximetry were monitored and recorded. Ventilator settings were adjusted to maintain a PaCO_2_ between 35 and 50 mmHg.
  d. Pulmonary vascular resistance was calculated as follows:

PVR = (mean PA pressure − LA pressure)/pulmonary blood flow in (mL·min^−1^·kg^−1^)

In lambs without PPHN, the left pulmonary arterial flow was used to calculate the “left” PVR. In lambs with PPHN, the main pulmonary arterial flow was used for this calculation.

## 3. Results

**Figure 2:**
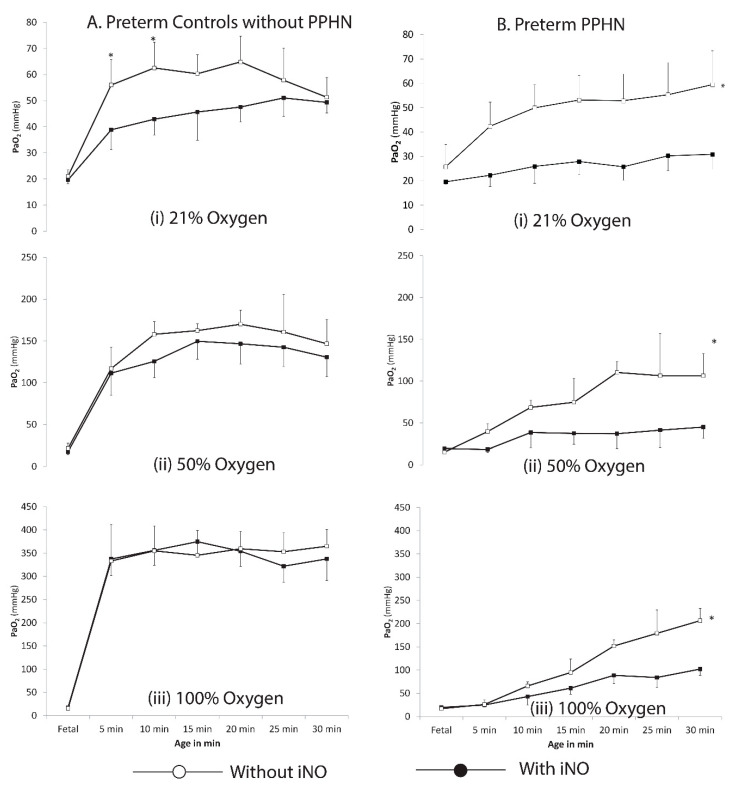
Changes in oxygenation with inhaled nitric oxide (iNO): PaO_2_ in the first 30 min of life in (**A**) preterm lambs without persistent pulmonary hypertension of the newborn (PPHN) with exposure to 21, 50 and 100% oxygen with (open squares) and without iNO at 20 ppm (solid squares). (**B**) PaO_2_ in the first 30 min of life in preterm lambs with PPHN on exposure to 21, 50 and 100% oxygen with (open squares)and without iNO (solid squares) at 20 ppm. * *p* < 0.05 compared to PaO_2_ without iNO.

**Figure 3:**
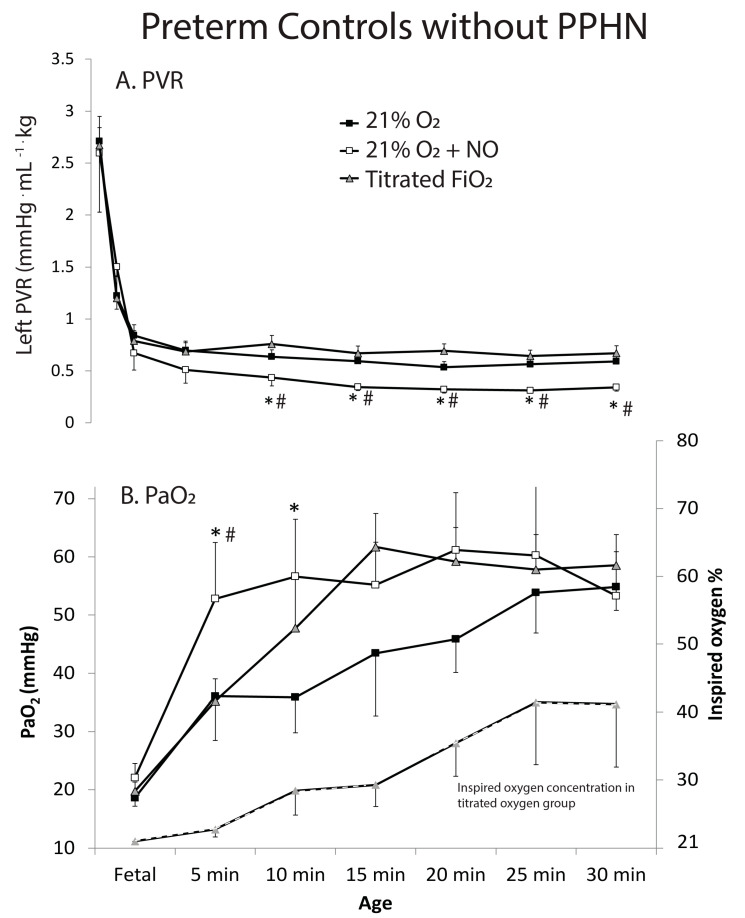
Changes in Pulmonary vascular resistance (PVR) in left lung (**A**) and PaO_2_ (**B**) in preterm lambs exposed to 21% oxygen (solid squares) vs. 21% oxygen and iNO (open squares) and titrated oxygen (gray triangles). Inspired oxygen concentration needed in the titrated oxygen group to maintain PaO_2_ between 45 to 80 mmHg is shown by a hyphenated line on the secondary *y*-axis (gray triangles). * *p* < 0.05 compared to 21% oxygen group; # *p* < 0.05 compared to titrated oxygen group.

**Figure 4:**
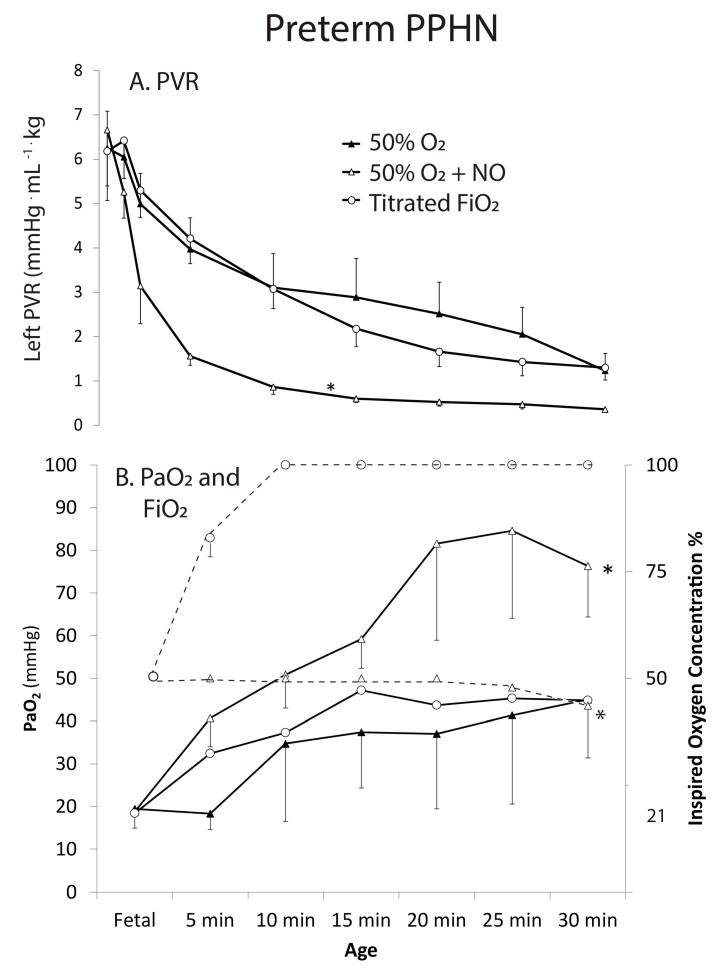
Changes in total pulmonary vascular resistance (PVR) in both lungs (**A**) and PaO_2_ (**B**) in PPHN lambs exposed to 50% oxygen (solid triangles) vs initiation with 50% oxygen and iNO and titration (open triangles) and titrated oxygen (open circles). Inspired oxygen concentration in the titrated oxygen group (open circles) and titrated oxygen with iNO (open triangles) is represented by a hyphenated line (* *p* < 0.05 compared to corresponding value without iNO.

I. **Oxygenation studies**: Thirty-six preterm lambs without PPHN and 30 lambs with PPHN were included in this phase of the study (6 lambs in each group). There was a significant difference in birth weight between preterm lambs without PPHN (3059 ± 105 g) and lambs with PPHN (2576 ± 210 g). The birth weights, gender distribution, and multiplicity were similar between the iNO and no-iNO groups (data not shown). Fetal blood gases were similar between preterm lambs with and without PPHN (PaO_2_ − 18.4 ± 7.2 and 17.5 ± 6.1 mmHg respectively) and between lambs ventilated with and without iNO (Figure 2).
  a. Preterm lambs without PPHN ventilated with 21% oxygen gradually increased their PaO_2_ over the first 30 min. Ventilation with iNO significantly increased PaO_2_ at 5 (39 ± 3 vs. 56 ± 11 mmHg) and 10 min (43 ± 3 vs. 63 ± 12 mmHg). There was no difference in PaO_2_ with and without iNO by 30 min (Figure 2Ai). Ventilation with 50 and 100% oxygen significantly increased PaO_2_ compared to 21% oxygen reaching supraphysiological levels by 5 min. However, addition of iNO did not increase PaO_2_ when preterm lambs were ventilated with 50 and 100% oxygen (Figure 2A).
  b. Preterm lambs with PPHN had low PaO_2_ values compared to lambs without PPHN (Figure 2). Ventilation with 21% oxygen resulted in low PaO_2_ values (30 ± 6 mmHg at 30 min). Increasing inspired oxygen from 21 to 50% significantly increased PaO_2_ in lambs with PPHN (45 ± 13 mmHg at 30 min with 50% oxygen). However, further increase in inspired oxygen from 50 to 100% did not further increase PaO_2_ (44.5 ± 20 mmHg at 30 min with 100% oxygen). Inhaled nitric oxide significantly increased PaO_2_ with 21, 50 and 100% oxygen in lambs with PPHN. Three PPHN lambs were hydropic (one each in 21% oxygen, 21% oxygen + iNO, and 50% oxygen groups) with massive pleural effusions and ascites and were excluded, reducing the number of lambs in these groups to five.
II. **Hemodynamic studies**: Fifteen preterm lambs without PPHN and 15 lambs with PPHN underwent thoracotomy and placement of pressure and flow probes to measure PVR.
  a. Preterm lambs without PPHN were divided into three groups. The first group was ventilated with 21% O_2_ (*n* = 5). A steady decline in PVR was measured with ventilation. The second group received titrated inspired oxygen adjusted every 5 min to maintain a PaO_2_ between 45 and 80 mmHg (*n* = 5). This required increase in inspired oxygen to 41 ± 9% by 30 min. The decline in PVR in this group was similar to the 21% oxygen group. The third group received iNO at 20 ppm and inspired oxygen was titrated to maintain PaO_2_ between 45 and 80 mmHg (*n* = 5). This group needed 21% oxygen throughout the 30 min period. The PaO_2_ and PVR with 21% oxygen + iNO was significantly lower than the previous two groups (Figure 3A,B). The preductal SpO_2_ value at 5 min was 66 ± 8, 63 ± 9 and 89 ± 11% in 21% O_2_, titrated O_2_ and 21% O_2_ + iNO groups respectively. The corresponding values at 30 min were 86 ± 11, 87 ± 10 and 85 ± 13%.
  b. Preterm lambs with PPHN were also divided into 3 groups. The first was ventilated with 50% oxygen (*n* = 5). There was a modest decrease in PVR and increase in PaO_2_ but some PPHN lambs remained hypoxemic with PaO_2_ < 45 mmHg (Figure 4). The second group received titrated inspired oxygen starting at 50% and adjusted to maintain PaO_2_ between 45 and 80 mmHg (*n* = 5). By 10 min, all lambs in this group were on 100% O_2_. The reduction in PVR and increase in PaO_2_ in this group was similar to the 50% oxygen group in spite of the significantly higher inspired oxygen. The third group was initially ventilated with 50% oxygen with iNO 20 ppm (*n* = 5). The PVR in this group was significantly lower and PaO_2_ significantly higher than the other two. By 30 min, inspired oxygen could be weaned to 44 ± 2% (Figure 4). The preductal SpO_2_ value at 5 min was 33 ± 14, 61 ± 11 and 71 ± 13% in 50% O_2_, titrated O_2_ and 50% O_2_ + iNO groups respectively. The corresponding values at 30 min were 78 ± 13, 80 ± 14 and 92 ± 8%.

## 4. Discussion

In the current study, we demonstrated that supplementation with iNO during resuscitation at birth in preterm lambs with and without PPHN reduced PVR and inspired oxygen concentration necessary to achieve target PaO_2_ levels. These findings have implications for delivery room resuscitation of preterm infants [10,11,12] and infants with PPHN [13].

The main goal of neonatal resuscitation is to achieve adequate ventilation of the lung and establishment of lung as the organ of gas exchange [5]. While 21% oxygen is effective for resuscitating term infants, the optimal oxygen concentration for resuscitation of preterm infants continues to be controversial [14], resulting in variations in clinical practice guidelines [15]. A comparison of low oxygen (initial oxygen concentration 21–30%) and high oxygen (60–100%) strategies has not demonstrated improvement in long-term outcomes in preterm infants. [3] Low-oxygen strategies are associated with reduced oxidative stress [16] and improved pulmonary outcomes in some studies [17]. However, not achieving a saturation of 80% by 5 min of postnatal age (whether due to inadequate oxygen supplementation or pulmonary or pulmonary vascular disease) is associated with adverse outcomes [12]. One approach to improving systemic oxygenation and promoting pulmonary vasodilation during transition at birth, while establishing the lung as the organ of gas exchange without excessive supplemental oxygen, is to use a selective pulmonary vasodilator such as iNO in the delivery room. In a recently published pilot double-blind randomized controlled trial on the use of inhaled nitric oxide in the delivery room resuscitation of extremely low birthweight preterm infants by Sekar et al., those who received 20 ppm iNO as an adjuvant in the resuscitation gas had a lower cumulative FiO_2_ exposure and a lower rate of exposure to FiO_2_ > 0.6 [18].

Inhaled NO improved PaO_2_, and reduced the need for supplemental oxygen from 41 ± 9 to 21% in preterm lambs. PVR significantly decreased with the use of 21% oxygen with iNO. Studies in extremely preterm infants have suggested increased mortality in infants resuscitated in 21% [4,19] Although the physiological basis of the increased mortality is not fully understood, one plausible explanation is that infants who were resuscitated in 21% oxygen were exposed to hypoxia and inadequate pulmonary vasodilation in the immediate newborn period. Our study demonstrated improved oxygenation by 5 min and improved pulmonary vasodilation with the use of iNO at birth in preterm lambs.

During fetal life, adequate oxygen delivery is achieved by an umbilical venous pO_2_ of 32–35 mmHg [20], and fetal PVR is high with physiologic pulmonary hypertension [21]. However, in the immediate postnatal period, similar PaO_2_ values resulted in hypoxic pulmonary vasoconstriction [22]. Achieving a preductal PaO_2_ of 45 mmHg (equivalent to 80% SpO_2_) by 5 min [12] is an important goal and can be achieved with lower supplemental oxygen if iNO is started at birth in preterm infants (Figure 1). This PaO_2_ of 45 mmHg is also the change point below which hypoxic pulmonary vasoconstriction was observed in newborn calves [22] and lambs [8,23]. Interestingly, the improvement in PaO_2_ in preterm lambs with iNO was observed only in the 21% oxygen group, not in the 50 and 100% groups. We speculate that the increase in alveolar oxygen (PAO_2_) and supraphysiological arterial oxygenation (PaO_2_) achieved with 50 and 100% inspired oxygen in preterm lambs induced pulmonary vasodilation and the addition of iNO did not result in further pulmonary vasodilation. The administration of 21% oxygen with iNO results in low PVR with the benefit of avoiding hypoxia without increasing the risk of hyperoxia.

In the lambs with PPHN, resuscitation with supplemental oxygen alone (including 100% oxygen) did not achieve optimal PaO_2_ levels by 5 and 10 min of postnatal age. Inhaled NO improves oxygenation at all levels of inspired oxygen (21, 50 and 100%). The use of iNO in the delivery room in infants with suspected PPHN may not be practical. In most cases, with the exception of congenital diaphragmatic hernia (CDH), PPHN is not diagnosed in the delivery room. In infants with CDH, iNO has not been effective in reducing the need for ECMO [24], and initial resuscitation with 50% oxygen is feasible and not associated with adverse events [13].

Preterm neonates have deficient antioxidant systems and are susceptible to oxygen toxicity [25]. Increased oxygen tension in the blood and tissues increases the risk of oxygen toxicity [26] by the formation of reactive oxygen species exceeding the antioxidant capability of the neonate [27]. The optimal inspired oxygen concentration should deliver an adequate amount of oxygen to the tissues at the lowest possible oxygen tension.

The use of high concentrations of oxygen or iNO at birth can have both short-term and long-term negative consequences. High concentration of oxygen during the resuscitation of an asphyxiated neonate can increase superoxide anions, [28] peroxynitrite and isoprostanes [9,29]. A combination of iNO and oxygen may have other unknown side effects (including potential epigenetic changes) [30,31]. The use of iNO in preterm infants with hypoxemic respiratory failure and pulmonary hypertension is controversial. [32,33,34,35] When used with high concentrations of oxygen, iNO can increase nitrosative stress. [9] Using iNO adds a significant risk of generating toxic nitrosative derivatives such as nitro-tyrosine, nitro-albumin and highly toxic perioynitrite. Nitric oxide scavenges superoxide anions by competing with superoxide dismutase. Superoxide anions are generated during the fetal-to-neonatal transition, especially when supplemental oxygen is provided [28]. Sequestration of superoxide by NO may lead to an apparent reduction in oxidative stress markers. [9] Simultaneous evaluation of nitrosative stress markers should be performed to avoid drawing erroneous conclusions. Inhaled NO use in the NICU has been associated with increased childhood malignancies [31,36]. Vento and Sanchez-Illana in a comment to the Sekar et al. trial recommend a long-term neurodevelopmental follow-up among preterm neonates exposed to iNO at birth [37].

There are several limitations to the current study. All preterm lambs were intubated during resuscitation. The effectiveness of iNO when administered through non-invasive ventilation in the delivery room is not known. However, in the pilot trial by Sekar et al., iNO was effective in reducing FiO_2_ during resuscitation in the delivery room [18]. We monitored pulse oximetry but mainly relied on frequent preductal PaO_2_ measurements for titrating inspired oxygen due to the variable relationship between SpO_2_ and PaO_2_ [38]. Such titration is not feasible in a clinical situation. Given the high concentration of fetal hemoglobin at birth, reliance on PaO_2_ may result higher risk of hyperoxemia compared to titration using SpO_2_. Preterm lambs were at 134 days gestation which corresponded approximately to late preterm infants at approximately 34 weeks gestation in late saccular stage. Lambs at 80 to 120 days of gestation corresponded to 17–27 weeks of human gestation and were in the canalicular stage of lung development [39]. This maturity at 134 days was older than the gestation of infants in the Sekar et al. trial (25–31 weeks) [18]. The ductal ligation model of PPHN did not have parenchymal lung disease. The results of this study may not be applicable to PPHN secondary to parenchymal diseases such as respiratory distress syndrome, meconium aspiration syndrome and pneumonia. Inhaled NO improved oxygenation in neonatal models of atelectasis [40], but its effectiveness in preventing hypoxic pulmonary vasoconstriction in such models is not known. It is possible that lower doses of iNO (<20 ppm) or other inhaled pulmonary vasodilators such as prostacyclin analogs may show similar results [41]. Presence of respiratory or metabolic acidosis may modify the lamb’s response to hypoxia. We did not study the effect of pH and asphyxiation at birth on oxygenation and response to iNO [22,42]. There are substantial differences between animal models and controlled translational studies and human neonates in a hectic clinical setting. Recent studies have shown stark differences in the outcomes of translational and clinical studies, such as sustained inflation in preterm infants at birth [43]. Lastly, we did not measure oxidative or nitrosative stress.

## 5. Conclusions

We concluded that iNO is effective for improving oxygenation in both preterm lambs and lambs with PPHN without increasing inspired oxygen. Inhaled NO reduced PVR in the immediate newborn period in preterm neonatal lambs with normal lungs and with PPHN. A randomized controlled masked pilot study evaluating the use of iNO in preterm infants showed a reduced FiO_2_ requirement with iNO use. Larger randomized studies with long-term follow-up and studies evaluating oxidative and nitrosative stress following short uses of iNO are warranted. The limited available information on the use of iNO in the delivery room precludes its use in neonatal resuscitation except in well-controlled trials.

## Data Availability

Data provided on request.
